# Stability effects on results of diffusion tensor imaging analysis by reduction of the number of gradient directions due to motion artifacts: an application to presymptomatic Huntington’s disease

**DOI:** 10.1371/currents.RRN1292

**Published:** 2012-02-02

**Authors:** Hans-Peter Müller, Sigurd D. Süssmuth, G. Bernhard Landwehrmeyer, Albert Ludolph, Sarah J Tabrizi, Stefan Kloppel, Jan Kassubek

**Affiliations:** ^*^Dept. of Neurology, University of Ulm, Ulm, Germany; ^¶^Department of Neurodegenerative Disease, UCL Institute of Neurology, London, UK and ^#^Freiburg Brain Imaging, Department of Psychiatry and Psychotherapy. University of Freiburg

## Abstract

In diffusion tensor imaging (DTI), an improvement in the signal-to-noise ratio (SNR) of the fractional anisotropy (FA) maps can be obtained when the number of recorded gradient directions (GD) is increased. Vice versa, elimination of motion-corrupted or noisy GD leads to a more accurate characterization of the diffusion tensor. We previously suggest a slice-wise method for artifact detection in FA maps. This current study applies this approach to a cohort of 18 premanifest Huntington’s disease (pHD) subjects and 23 controls. By 2-D voxelwise statistical comparison of original FA-maps and FA-maps with a reduced number of GD, the effect of eliminating GD that were affected by motion was demonstrated.

We present an evaluation metric that allows to test if the computed FA-maps (with a reduced number of GD) still reflect a “true” FA-map, as defined by simulations in the control sample. Furthermore, we investigated if omitting data volumes affected by motion in the pHD cohort could lead to an increased SNR in the resulting FA-maps.

A high agreement between original FA maps (with all GD) and corrected FA maps (i.e. without GD corrupted by motion) were observed even for numbers of eliminated GD up to 13. Even in one data set in which 46 GD had to be eliminated, the results showed a moderate agreement.

## **Introduction**

White matter (WM) changes in Huntington’s disease (HD) patients´ brains have already been shown in a number of studies (see [1], [2] for reviews) and have mainly been studied using Diffusion Tensor Imaging (DTI) [3],[4]. DTI as an advanced approach to WM analysis is relatively robust against movement artifacts and some other sources of noise when only single volumes are affected. Motion artifacts might lead to noisy results in the neuroimaging research of neurodegenerative disorders such as dementing diseases or hyperkinetic movement disorders, e.g. HD. There is no systematic effect of movement on FA-values in FA-maps, i.e. FA-values can either increase or decrease as a result of movement artifacts. Therefore, the effect of motion artifacts on the resulting analysis has to be carefully considered in order to provide biomarkers useful for clinical trials [2],[5].

DTI, as one application of diffusion weighted imaging (DWI), characterizes the combination of diffusion directions in each voxel and is based on the diffusion of water which is influenced by local tissue properties, since DWI-sequences combine several gradient directions (GD), each of which codes the diffusion along its direction [6]. The shape information of the resulting tensor can be converted into compound measures such as fractional anisotropy (FA – [7],[8]), in order to allow comparisons at the group level and to correlate clinical markers with imaging.

In general, any measured signal is a combination of the true quantity, the acquisition system noise, the environmental noise and the subject specific noise:

\begin{equation*}S_{measured} =S_{true} +N_ {acquisition System} +N_{environment} +N_{subject}\end{equation*}

                                        (1)

Ideally, the measured signal is a sum of the true quantity and ideal noise,

\begin{equation*}S_ {measued} =S_{true} +N_{ideal}\end{equation*}

                                                                                                                                    (2)

so that the quality of the measured signal could be improved by signal accumulation, and the SNR condition

\begin{equation*}SNR\sim \sqrt{n}\end{equation*}

                                                                                                                                                                          (3)

holds, where _\begin{equation*}n\end{equation*}  _ denotes the number of signal accumulations [9]. 

If these considerations were transferred to calculations from DTI acquisitions where the required minimum number of GD is 6 due to the 6 independent components of the diffusion tensor ***D***, an SNR improvement in the tensor estimation and subsequently in the FA calculation is reached by an increased number of GD [10]. Also a more robust fiber tracking (FT) could be obtained by an increasing number of recorded GD. The condition of Equation 3 does not hold when e.g. subject movements contribute to the measured signal. In this case, the contribution of such corrupted volumes leads to a decrease of the SNR [9].

Thus, if a certain number of volumes, i.e. GD, are corrupted by subject movement or other sources of noise, a decrease in SNR is the result of integrating such corrupted volumes into the tensor calculation. An improvement in SNR could be obtained by omitting noisy or corrupted volumes from the tensor calculation. In a previous study [5], we introduced a method to detect and eliminate volumes corrupted by motion from a given DTI data set: a framework for the identification of these volumes was introduced as an instrument of quality control (QC), and a suggestion was given which volumes should be omitted for tensor calculation [5]. A further comprehensive analysis of error in FA estimation was reported by Liu et al. [11]. The question has to be addressed what the exact effect on the resulting FA-maps and the FT is if a certain number of GD remain unused for tensor calculation.

Farrell et al. [12] provided a single subject study as a framework for the effects of SNR on the accuracy and reproducibility of DTI-derived parameters. The aim of the current study was to demonstrate the effects of omitting volumes in a cohort of healthy controls, and to develop an algorithm if, after omitting volumes, the calculated FA-maps represent the “true” FA-map according to Equation 1 (i.e. the method is appropriate for increasing the SNR of the remaining FA-values).  

## **Methods**

### Data recording and subjects

Eighteen pHD subjects and 23 age-matched controls were scanned on a single 3 *Tesla* scanner (TRIO, Siemens, Erlangen, Germany). The sequence consisted of 72 diffusion-weighted imaging scans, each with dimensions of 96 pixels x 96 pixels x 55 slices per volume with a 2.3 mm isotropic voxel size; 64 unique diffusion gradient directions (b = 1000 s/mm^2^) and eight b = 100 s/mm^2^ images; TE was 90 ms. The MRI scans were acquired as part of the London site TRACK-HD cohort (see also acknowledgment). 

### Ethics Statement

The study was approved by the Ethics committee of the University of Ulm, and written informed consent was obtained from each subject. The authors' responsible institutional review boards approved the study.   

### Quality Check (QC)

In case of motion disturbances during the acquisition, i.e. in case of corrupted volumes, an SNR increase is obtained by omitting single GD for tensor calculation. For that purpose, we have adopted the previously developed QC algorithm [5]. In brief, for scans that contained corrupted volumes, attempts to increase SNR were made by omitting single gradient directions one at a time before tensor estimation: for each GD, the weighted variance was computed from all remaining directions in the sequence by weighting with the angle in which they differed from the index GD [5]. 

An artifact correction was performed by detecting GD with at least one slice showing decreased intensity, i.e. motion artifacts caused by spontaneous subject movement. For any diffusion weighted volume, the mean intensity for each slice was computed and its intensity was compared with the same slice in all other volumes by using a weighted average approach – the weighting factor was the dot product of vectors of two GD \begin{equation*} \vec  {g}_i \vec  {g}_j\end{equation*}:

_\begin{equation*}\Delta _j = 1 - \frac{1}{N}  \sum_{0}^{N}  \vec  {g}_i \vec  {g}_j\Delta I_{ij}\end{equation*}_ ,                                                                                                                                (4)

where

\begin{equation*} \Delta I_{ij}} =\frac{\left| <a_i> - <a_j> \right|}{<a_i> + <a_j>}\end{equation*}.

                                                                                                                                                  (5)

_\begin{equation*}<a_i>\end{equation*}_ denotes the arithmetic average intensity of the slice under observation and a slice for comparison. The relative average intensity deviation \begin{equation*}\Delta I_{ij}\end{equation*} was weighted by the dot product of the GD. Thus, in order to define a global parameter for DTI QC:

\begin{equation*}Q = min(I_j)_i\end{equation*}

                                                                                                                                                               (6)

reflects the minimum of slicewise comparisons of all slices. If ***Q*** undergoes a certain threshold, the whole volume (i.e., GD) was eliminated. A threshold of 0.8 was suggested as a stable solution  [5]. Nevertheless, in order to improve precision, a more conservative threshold might be chosen. In other words, Equations 4-6 detect volumes where single slices show low intensity due to subject motion compared to slices of other volumes weighted by the GD orientation. Further details are presented in [5].    

### Effect of reducing the number of GD

In the current study, the methodological framework was used for the first time to define a decision metric for the application in group studies. Calculation of all possible permutations of omitted or used GD, respectively, for tensor calculation is in the order of hundreds of millions of possibilities. Therefore, an exemplary approach was used, i.e. a set of randomly selected GD was omitted and FA-maps were calculated for the original data set (72 GD including 8 b=0 scans) \begin{equation*}FA_{orig}\end{equation*} as well as for data subsets with a reduced number of GD (*N* GD including 8 b=0 scans) _\begin{equation*}{FA}_{red}\end{equation*}_. In order to investigate the effect of omitting GD on FA-maps, for the data of the healthy controls, the FA-maps and were statistically compared by Student's t-test to obtain a p-value as a metric for the difference. Statistical comparison included all FA-values above 0.2 - an FA-value of 0.2 has already been suggested as an appropriate threshold in FA analyses [13], [14].

In the following, the effect of motion corrupted volumes on the FA maps was investigated. Since single FA-values could be more or less influenced by single corrupted GD, the influence on the whole FA map (or a certain ROI in the FA map) was analysed for that purpose. The data analysis was performed in two ways: First, randomly selected GD were omitted in the control data sets and the effect on the FA-maps was calculated, where

_\begin{equation*}p (FA_{orig} ,FA_{red} ) = sign(ttest ( FA_{orig}, FA_{red} ) )\end{equation*}_                                                                                        (7)

denotes the p-value for a statistical comparison of the FA-maps by t-test. This metric gives a quantitative parameter as an estimate of the effect on FA maps if a certain number of GD (that do not contribute to the SNR improvement) is omitted.

In a second step, instead of randomly excluding GD, the GD that had been identified as corrupted by the QC (Equations 5 and 6) in the pHD cohort were omitted in the control data sets and the corresponding p-values were calculated (Equation 7). That way, the effect of eliminating corrupted volumes corrupted by head movements (e.g. in data sets of pHD subjects) could be estimated in the control data set and thus could act as decision criterion if the whole 3D-DTI data set passed QC.

That way, the improvement of SNR by defined GD was estimated in a sample of control data sets. Thus, the effect on FA maps in the control data set sample could act as an estimator for the effect on the SNR improvement in pHD data sets where the motion corrupted volumes were eliminated.

If a low influence by additional sources of noise was assumed (as it should be the case for control subjects), i.e. Equation 2 is valid rather than Equation 1, then Equation 3 might be used for estimation, i.e. SNR is increasing with increasing number of GD. On the other hand, motion corrupted GD lead to a reduction of SNR (Equation 1) so that elimination of these motion corrupted GD avoids this reduction of the SNR.  

**Figure:**
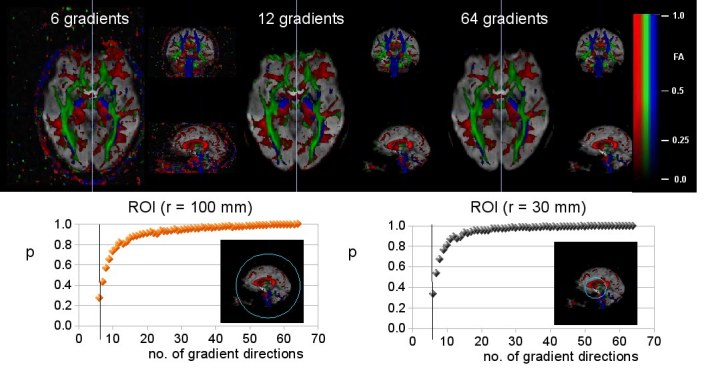
**Figure 1:** **Upper panel:** Examples of fractional anisotropy (FA)-maps of a healthy control (c03) with arbitrarily selected gradient directions (GD) (6, 12, and 64, respectively) are displayed. Note the increase noise level in images based on 6 GD only. **Lower panel:** Dependence of the p-value (as an indicator for the signal-to-noise ratio) on the number of used GD for all voxels with an FA-value > 0.2 for the whole brain (**left**) and for a region of interest (r = 30mm) including the striatum (**right**) [15].

## **Results**

Figures 1 and 2 show that the p-value depended on the number of used GD for all voxels with an FA-value > 0.2, both for the whole brain (left) and for a ROI with r = 30mm (right) which included the striatum and was thus considered to be sensitive for affectation by HD-associated neurodegeneration  [15]. In order to show the effect of the eliminated GD on the FA, one healthy subject data set (control01) that showed a high DTI-QC value (> 0.9 for all volumes) was selected for exemplary FA-map comparison. In Figure 1, upper panel, examples of FA-maps are displayed. The FA-maps calculated on the basis of a lower number of GD show a lower SNR than those which were calculated with inclusion of all GD, as expressed in terms of p-values from statistical comparison of FA-maps. Each simulation of GD elimination was performed by averaging six GD sets (Figure 1, lower panel): the p-values are above 0.8 if more than 10-15 GD were used for calculation, i.e. in average a good coincidence between original FA-maps and FA-maps calculated from a reduced number of GD could be shown for total volume comparison (Figure 1, lower panel, left) as well as for the above-named ROI (Figure 1, lower panel, right).

**Figure:**
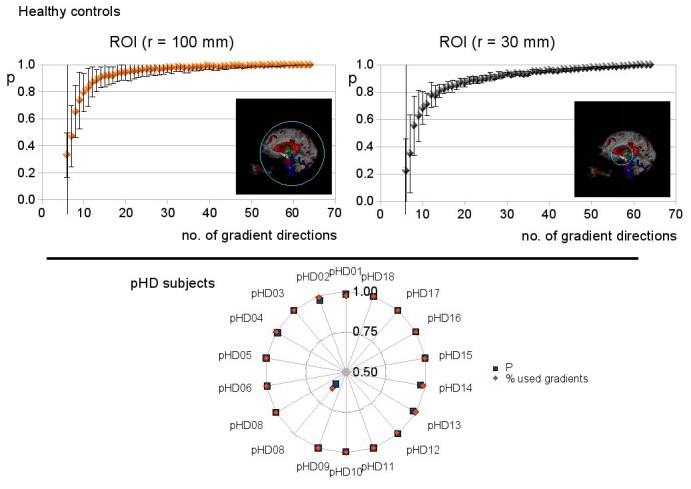
**Figure 2:** **Upper panel:** Relation between used gradients and the p-value differences between the FA-maps. Data were averaged for simulations of GD eliminations in 23 controls, both for the whole brain (**left**) and for the ROI (r=30mm) as depicted in Figure 1 (**right**). **Lower panel:** p-values (Equation 7) indicating differences between whole brain FA-maps with all GD and corrected FA-maps that were calculated with a restricted number of GD (using Equation 6 for quality control) and percentage of used GD for the premanifest HD (pHD) subjects where a certain number of GD was eliminated due to motion artifacts. The p-values and the proportion of used GD for each subject were arranged in a circle in order to express that there is no ranking in the labeling of the controls.

P-value results of the 23 healthy controls after random elimination of GD were arithmetically averaged. In the analysis of the relation between gradients used and the p-value differences between the FA-maps, the p-values exceeded 0.8 if more than 10-15 GD were used for calculation both for the whole brain and the selected ROI, so that the results were in accordance with the results in the pHD sample (Figure 2, upper panel). However, since the standard deviations were high for a low number of used gradients (and low for a high number of gradients, respectively), p-values for single data sets could be much higher or lower than these average values. The main effect is that different GD were selected by the random number generator for the data sets. 

Calculating the p-values (at whole brain level) for the pHD subjects where a certain number of GD was eliminated due to motion artifacts, a high agreement between original FA maps (with all GD) and optimized (i.e. without GD affected by motion) FA maps were observed (Figure 2, lower panel), with the exception of one data set. Nevertheless, from this calculation alone, it could not be stated if the recorded FA values or the optimized FA maps do better represent “true” FA-maps. 

In order to get a criterion of the influence of the eliminated GD on the FA calculation, the use of representative data sets is suggested. Twenty-one healthy volunteer data sets that showed a high DTI-QC (> 0.8 for all volumes, i.e. no volume was omitted for FA-calculation) were selected for this approach. These data sets could act as the reference for setting up a criterion for eliminating GD. The FA-maps calculated on the basis of a lower number of GD show a lower SNR than those calculated with inclusion of all GD.

**Figure:**
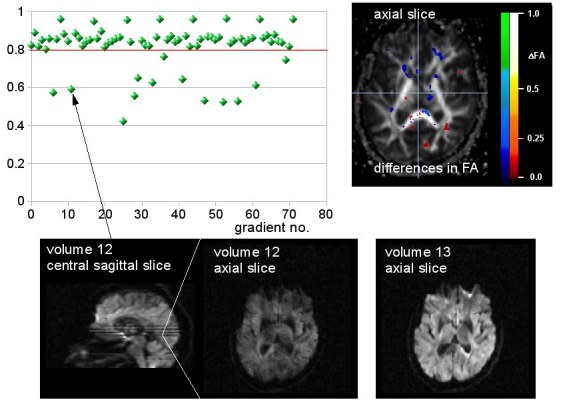
**Figure 3:** QC of the data set of a presymptomatic HD (pHD) subject (with slight head motions during the acquisition). **Upper panel, left:** With a QC-threshold of 0.80, 13 directions were eliminated.**Upper panel, right:** Differences in FA-maps (72 directions and 59 directions, respectively) with a p-value of 0.984 for whole brain. **Lower panel: **Representativecentral sagittal slice where axial slices affected by noise are visible (**left**). For comparison reasons, the same axial slice of another volume is shown (**right**). 

In Figure 3, upper panel, a representative data set of a pHD subject (with slight head motions during the acquisition – pHD01) is shown. QC suggested the elimination of 13 GD when a threshold of 0.8 was applied. The artifacts in axial direction are visible in sagittal slices (lower panel, left); for comparison, the same axial slice of another volume is shown (lower panel, right). Differences in FA-maps (64 directions versus 51 directions) were displayed in Figure 3, upper panel, right, with a p-value of 0.997 for the whole brain and 0.974 for the 30 mm ROI around the basal ganglia.

Now, for all pHD subjects the GD that were identified to be eliminated by QC (Equations 2, 3), were omitted to calculate in all 21 selected control data sets. In Table 1, averaged p-values of all control data sets ( and ) were summarized for the 11 pHD subjects where elimination of a particular GD number was necessary.

Figure 4 illustrates the p-values of the FA-map comparison for all control data sets in which the GD of the pHD were omitted. Although a defined number of GD was omitted, a high p-value for all control data sets could be found, indicating high coincidence between the FA-maps. Only the data set of subject pHD08 (with 46 GD eliminated) showed lower p-values (Figure 4).

In conclusion, all except one pHD data set (pHD08 who already was excluded in the previous work [5]) passed QC after elimination of GD. Thus, elimination of the GD in 10 of 11 pHD data sets led to an increase in SNR. Subject pHD08 (compare Table 1 and Figure 4) showed a low p-value comparing original FA-map and FA-map with 46 eliminated GD (Figure 2), whereas omitting the same GD in control data sets caused an average reduction to a p-value of 0.91.

**Figure:**
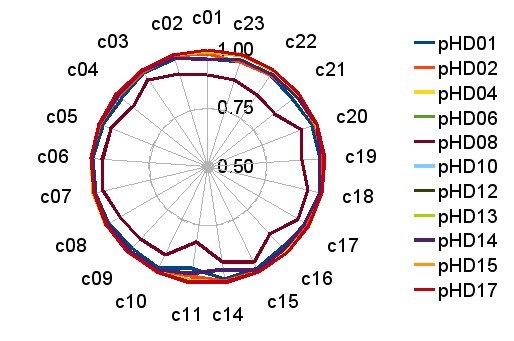
**Figure 4:** Plot of p-values for 21 selected control data sets (NB, controls 12 and 13 could not be used due to noise corrupted GD) where those GD were omitted that had been suggested for elimination in the respective presymptomatic HD (pHD) sets (indicated in colors). The p-value results for each control were arranged in a circle (for averaged p-values, refer to Table 1). For pHD08 (46 eliminated GD, brown line), the p-values form a circle indicating lower similarity for the FA-maps of the controls with 46 omitted GD, as compared to FA-maps with 72 GD.

**Table:** 

**subject**	**no. elim. GD **	** avg. p-value **
pHD01	13	0.98
pHD02	7	0.99
pHD04	2	1.00
pHD06	1	1.00
pHD08	46	0.91
pHD10	1	1.00
pHD13	1	1.00
pHD14	3	1.00
pHD15	13	0.98
pHD16	1	1.00
pHD19	1	1.00

**Table 1:** Averaged p-values derived from the selected control data sets where those GD were omitted that were suggested to be eliminated in the respective 11 presymptomatic HD (pHD) data sets (compare Figure 4).

## **Discussion and Conclusion**

Motion corrupted volumes could be the cause of a lower SNR in FA-values/FA-maps in DTI studies of subjects with limited compliance. A tool was developed to detect motion corrupted volumes in DTI data sets in order to eliminate these volumes prior to FA-calculation. This study showed the advantages of omitting volumes in FA-calculation , thus providing a framework to estimate the effect of eliminating volumes in DTI data sets, i.e. the identical sample of GD (that were detected as motion corrupted by QC) was omitted in DTI data sets from controls in order to provide a metric for the effect on the SNR. This study shows a high stability of FA-results in cases when a certain number of GD has been omitted. These findings are in accordance with DTI studies making use of e.g. 12 or 30 GD (see e.g.  [16]). Therefore, the changes in FA values without and with QC, respectively, will be low for single data sets if the relation between eliminated volumes and the total number of volumes is also low. In this case, QC could act as a tool to improve the quality of single subject results and consequently to improve the results at the group level. Subsequent group comparisons could compensate for the remaining outliers. This QC detection method is suitable for DTI sequences that employ a high number of unique directions rather than scanning fewer directions multiple times. 

The difference of FA-maps with and without eliminated GD lacked reference data and thus could not act as decision criterion, i.e. it could not be decided if a decrease or an increase in SNR is achieved by eliminating GD. For this task, healthy volunteer data sets could be used as a common reference if a certain number of volumes have been identified by QC and are to be omitted in subsequent analyses. 

This study extends previous DTI postprocessing analysis [5] where we introduced an objective criterion to check the validity of results if a defined number of GD is omitted. The aim is to obtain FA-values in studies at the group level that have the same data quality for all subjects. The threshold effectively controls the trade-off between an unnecessary loss of data by being too conservative and the inclusion of too much noise by applying too lenient thresholds. A threshold between 0.7 and 0.8 led to reasonable results in  [5], but could be adapted for other studies to reach more sensitive results. For instance, a study at the group level with advanced HD patients (when a lot of movements more might be expected) should define a lower threshold so that more volumes could contribute to the signal.

In this study, exemplary eliminations have been performed. Nevertheless, these examples are to be considered representative if the eliminated, or consequently the residual, GD have a homogeneous directional distribution. Any elimination has to be checked separately as special elimination configurations could lead to stronger effects. Although excessive movement and other artifacts increase noise and therefore decrease the sensitivity to detect true artifacts, the method is encouraging for large-scale studies in diseased subjects, since DTI is a promising biomarker in the research of neurological diseases [17]. FA values are relatively robust to such kind of disturbances, and systematic errors could be excluded. When the QC tool will be included into the postprocessing, this might possibly allow for a higher flexibility in the analysis with respect to the applied threshold. In this approach, averaged p-values in relatively large ROIs were calculated - for strictly circumscribed artifacts, smaller ROIs might be investigated.

We are aware that the control data sets could not act as a gold standard per se. Nevertheless, with the method described in this work, the main difference between control data sets and pHD data sets, i.e. motion corruption of single volumes, could be reduced and thus data samples are brought to a level that is more comparable in group studies. For this task, a certain number of control data sets (showing motion artifacts) had to be removed for further analysis, i.e. the FA-map comparison for all control data sets in which the GD of the pHD were omitted (Table 1, Figure 4). If other sources of noise should be eliminated from DTI data sets, methods that are described in [11] can be used. The application of those methods can further improve data quality and SNR. 

## Funding information

This work was supported by the European HD network (EHDN project 070). The MRI scans were acquired as part of the London site TRACK-HD cohort. TRACK-HD is supported by the CHDI Foundation, a not for profit organization dedicated to finding treatments for HD.

## Acknowledgement 

The authors wish to extend their gratitude to the London TRACK-HD study participants and to Beth Borowsky, scientific director for TRACK-HD at CHDI. Some of this work was undertaken at UCLH/UCL who acknowledge support from the respective Department of Health's NIHR Biomedical Research Centres.****

## **Competing interests**

The authors have declared that no competing interests exist.
